# Supplementary material for: Potential use of lenvatinib for patients with unresectable hepatocellular carcinoma including after treatment with sorafenib: Real-world evidence and in vitro assessment via protein phosphorylation array
Source: Oncotarget. 2020 Jun 30;11(26):2531–42. doi: 10.18632/oncotarget.27640 (PMC7335665; doi:10.18632/oncotarget.27640)
Supplement: Supplementary file 1 [file oncotarget-11-2531-s001.pdf]

Potential use of lenvatinib for patients with unresectable hepatocellular carcinoma including after treatment with sorafenib: Real-world evidence and *in vitro* assessment via protein phosphorylation array

SUPPLEMENTARY MATERIALS

Preparation of cell lysates for protein array

PLC/PRF5 and PLC/PRF5-R2 cells were seeded at 2×10^6 cells/ml in 10% FBS and DMEM at 37°C and 5% CO_2. Cells were treated with 10 ng/ml LEN (LC laboratories, New Boston ST, Woburn, MA, USA) for 48 h. After treatment, the cells were washed twice with PBS. For phosphorylation analysis, the cells were lysed in M-PER (Thermo Fisher Scientific, Waltham, MA, USA) and supplemented with protease and phosphatase inhibitor cocktail (Thermo Fisher Scientific) following the manufacturer's instructions. The cell lysates were then centrifuged at 14,000 g for 10 min at 4°C and stored at –80°C until measurement.

Measurement of phosphorylation activity on the array

In order to investigate the degree of phosphorylation of constituent proteins in signal transduction pathways, proteins involved in signal transduction were surveyed with reference to "Signal Transduction" pathways at "Event Hierarchy" in the Reactome Pathway Database (https://www.reactome.org) and "Signal Transduction" pathways at "Environmental Information Process" in Kyoto Encyclopedia of Genes and Genomes (http://www.genome.ad.jp/kegg/) (KEGG).

Among the proteins checked, 1205 proteins belonging to 377 pathways in total were selected for detection. In addition, the 377 pathways were further categorized into 30 pathways for interpretation of biological functions. Degree of phosphorylation of selected proteins was determined by the protein array and the activity of the 377 pathways was estimated by a computational method (detailed below).

One thousand, two hundred and five genes in signal transduction pathways were synthesized using the WEPRO7240G (Cell Free Science, Ehime, Japan) wheat germ expression system. Synthesized proteins were then resuspended in PBS and 50 mM GSH solution (pH 7.3) in 50 mM Tris-HCl and spotted on glass slides (high-density APS slide SDM0011, Matsunami Glass) coated with 0.1 mM sSMPB (Thermo Fisher Scientific) using Genex Arrayer (Kaken Geneqs, Chiba, Japan). The lysate volume applied to the array was adjusted by the amount of total protein measured by BCA assay (ThermoFisher Scientific), and 100 µg of total protein was applied per slide. Slides were then stored at –80°C in blocking buffer (50 mM Tris-HCl [pH 7.3], 200 mM NaCl, 0.08% (v/v) Triton X-100, 5% phospho blocker, 5 mM GSH [pH 7.3], 25% (v/v) glycerol, and 2 mM DTT). After thawing at room temperature, kinase or lysate was suspended in Kinase Reaction Buffer (25 mM Tris-HCl [pH 7.5], 5 mM β-glycerophosphate, 0.1 mM Na_3VO_4, 10 mM $MgCl_2$, 1 mM ATP, and 2 mM DTT) and applied to the array plate for 18 h at 30°C. Kinase reaction was terminated by incubation with termination buffer (50 mM EDTA, 10 mM HEPES-NaOH [pH 7.4], 150 mM NaCl, and 0.05% [v/v] Tween 20) for 5 min at 30°C. Slides were then washed with 2x TBST washes for 5 min each. Phosphorylated tyrosine residues were detected by incubation with 4G10 platinum anti-phosphotyrosine (mouse monoclonal cocktail IgG2b) monoclonal antibody (Sigma-Aldrich), diluted 1:2,000 in 5% PhosphoBlocker in TBST, for 1 h at room temperature. After washing, secondary staining by goat anti-mouse IgG (H+L) secondary antibody conjugated with Alexa Fluor 647 (Thermo Fisher Scientific) in 5% PhosphoBlocker in TBST was performed for 1 h at room temperature. Array slides were then washed twice with TBST for 5 min each, TB buffer (20 mM Tris-HCl, pH 7.3) for 5 min, and distilled water for 5 min, and dried by centrifugation (800 g, 3 min). Fluorescence was detected by InnoScan scanner, and signal was calculated by Mapix (Innopsys, Chicago, IL). Finally, each signal was corrected for the corresponding background value of the plot output from Mapix. The principle of the protein array mentioned above is shown in Supplementary Figure 2.

Estimation of active pathway by protein array

We estimated the active pathways by two methods: “network screening”, which considers the coordination of phosphorylation degrees for whole constituent proteins with their connectivity in the pathway; and standard “pathway analysis”, which considers the number of proteins with the difference of phosphorylation degrees of proteins belonging to each pathway. The details of the two methods are as follows.

The “network screening” procedure is briefly described as follows [1]. The template of the network structure, the binary connections of proteins in the pathways, was first prepared for estimating the consistency between protein connectivity and the measured phosphorylation degrees of proteins. Since our method is applied to directed acyclic graph, we manually modified the original pathways in the Reactome and KEGG database, according to the following rules: 1) The directions of arrows were set from the proteins in the plasma membrane to those in the nuclear membrane; 2) In the phosphorylation of a protein by a complex of proteins, the arrows were assumed from each of the constituent proteins in the protein to protein complex; 3) In the pathway including a feedback loop, we separated one pathway into two pathways that were in forward and backward directions.

Finally, we constructed the template of 377 pathways for 1205 proteins.

In the next step, we calculated the graph consistency probability (GCP), which expresses the consistency of the phosphorylation degrees of the 1205 proteins on our array with each of the 377 pathway structures described by the binary connection template. The consistency of the pathway structure with the measured data is quantitatively expressed by the logarithm of the likelihood based on the Gaussian graphical model (GN: Gaussian Network) [2]. Since the likelihood depends on the graph size (numbers of nodes and edges), we designed a simple procedure to transform the likelihood into the probability (GCP) for simultaneous comparison between graphs with different sizes. Indeed, we generated N graphs under the condition that the graphs shared the same numbers of nodes and edges with the given graphs. Then, we simply defined GCP as the fraction of the number of graphs with larger log-likelihoods than the log-likelihood of the query graph to N. In the present study, Nr was set to 500.

Apart from the detection of the coordinately phosphorylated pathways above, we also estimated the difference in the phosphorylation degrees of the proteins by standard “pathway analysis”. The difference in the phosphorylation degree of each pathway between the two groups was estimated by Welch’s *t-test* ($p < 0.05$), and the number of proteins with significant probability was counted for each pathway. Then, the probability of each pathway was estimated based on the hyper-geometric distribution in the 377 pathways for the 1205 proteins. The thresholds of the probabilities were set to be 0.3 and 0.03 in the network screening and the pathway analysis, respectively.

Software

All statistical tests, with the exception of network screening, were performed using R software [3].

REFERENCES

1. Saito S, Aburatani S, Horimoto K. Network evaluation from the consistency of the graph structure with the measured data. BMC Syst Biol. 2008; 2:84. https://doi.org/10.1186/1752-0509-2-84. [PubMed]
2. Whittaker J. Graphical Models in Applied Multivariate Statistics. Wiley Publishing. 2009; 462.
3. Dag O, Dolgun A, Konar NM. onewaytests: An R Package for One-Way Tests in Independent Groups Designs. The R Journal. 2018; 10:175–99.

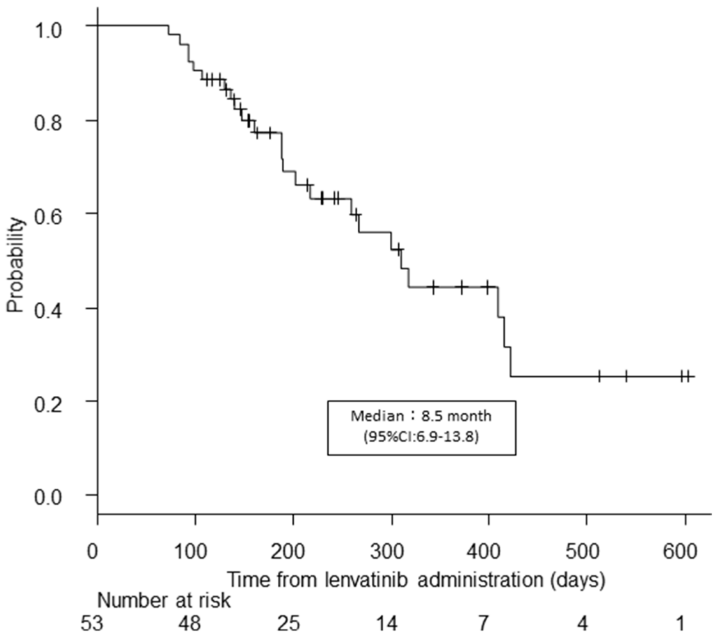

Supplementary Figure 1: Progression-free survival among patients with advanced hepatocellular carcinoma treated with lenvatinib.

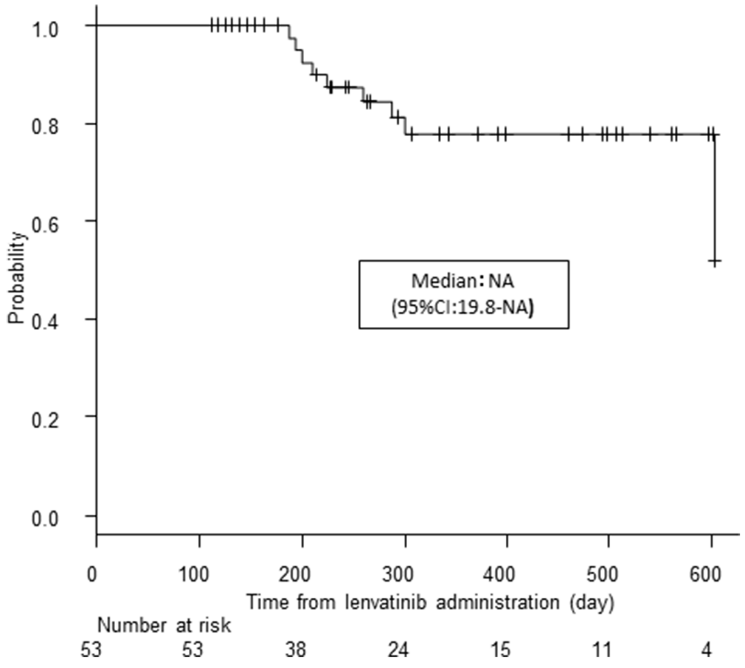

Supplementary Figure 2: Overall survival among patients with unresectable hepatocellular carcinoma treated with lenvatinib.

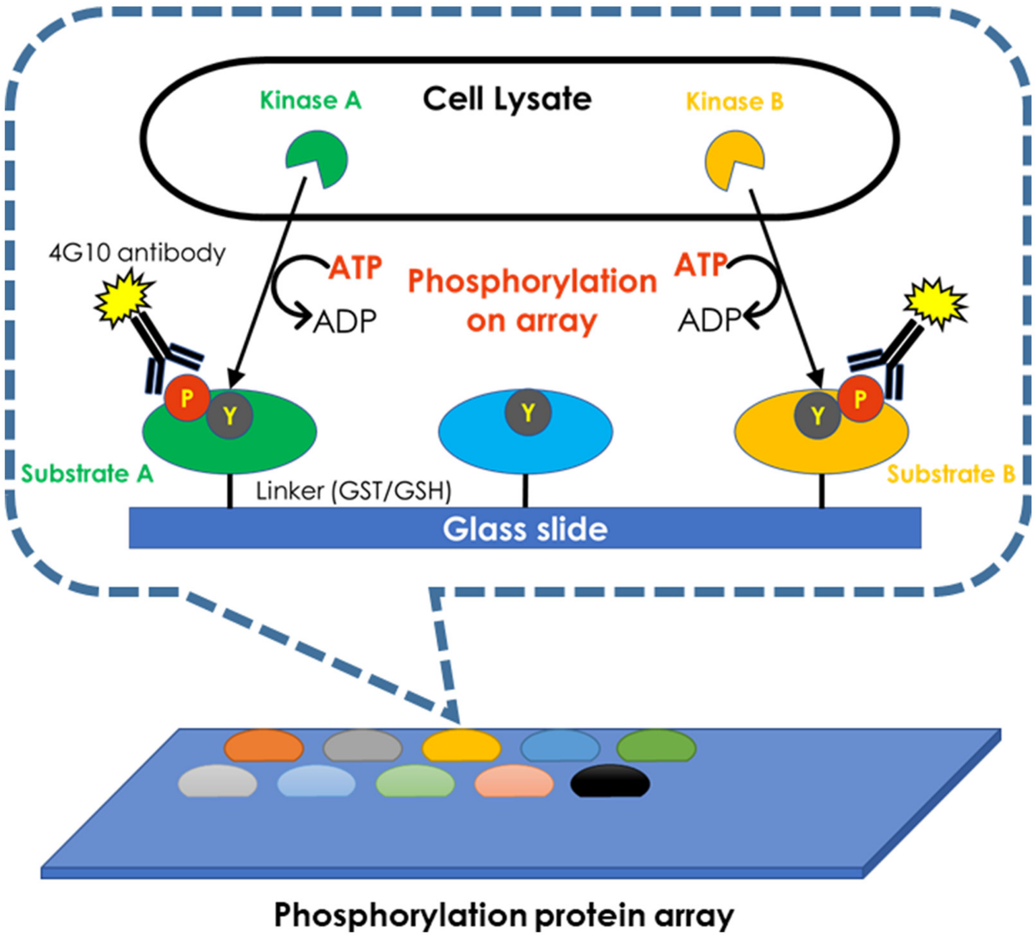

Supplementary Figure 3: Schematic representation of comprehensive protein phosphorylation profiling using the protein array. Kinase phosphorylation reactions in cell lysates were assayed on a protein array of 1205 proteins loaded on a GST/GSH-coated glass slide. Abbreviations in the figure are as follows: P, phosphate group; Y, tyrosine; ATP, adenosine triphosphate; ADP, adenosine diphosphate. Generally, in an antibody array used for phosphorylation analysis, the antibodies used are specific to one protein, and the binding of its specific protein is detected by an optic (fluorescence) device. Thus, the possible number of detected proteins are limited by the number of antibodies which specifically bind to proteins. In contrast, our original array was equipped with protein substrates phosphorylated by tyrosine kinases, and the degree of protein phosphorylation was detected by optic (fluorescence) device. Thus, all of the proteins that are possibly phosphorylated by tyrosine kinases in the lysate can be assessed, and in our array, the phosphorylation degrees of 1205 proteins belonging to 377 signal transduction pathways can be measured simultaneously.

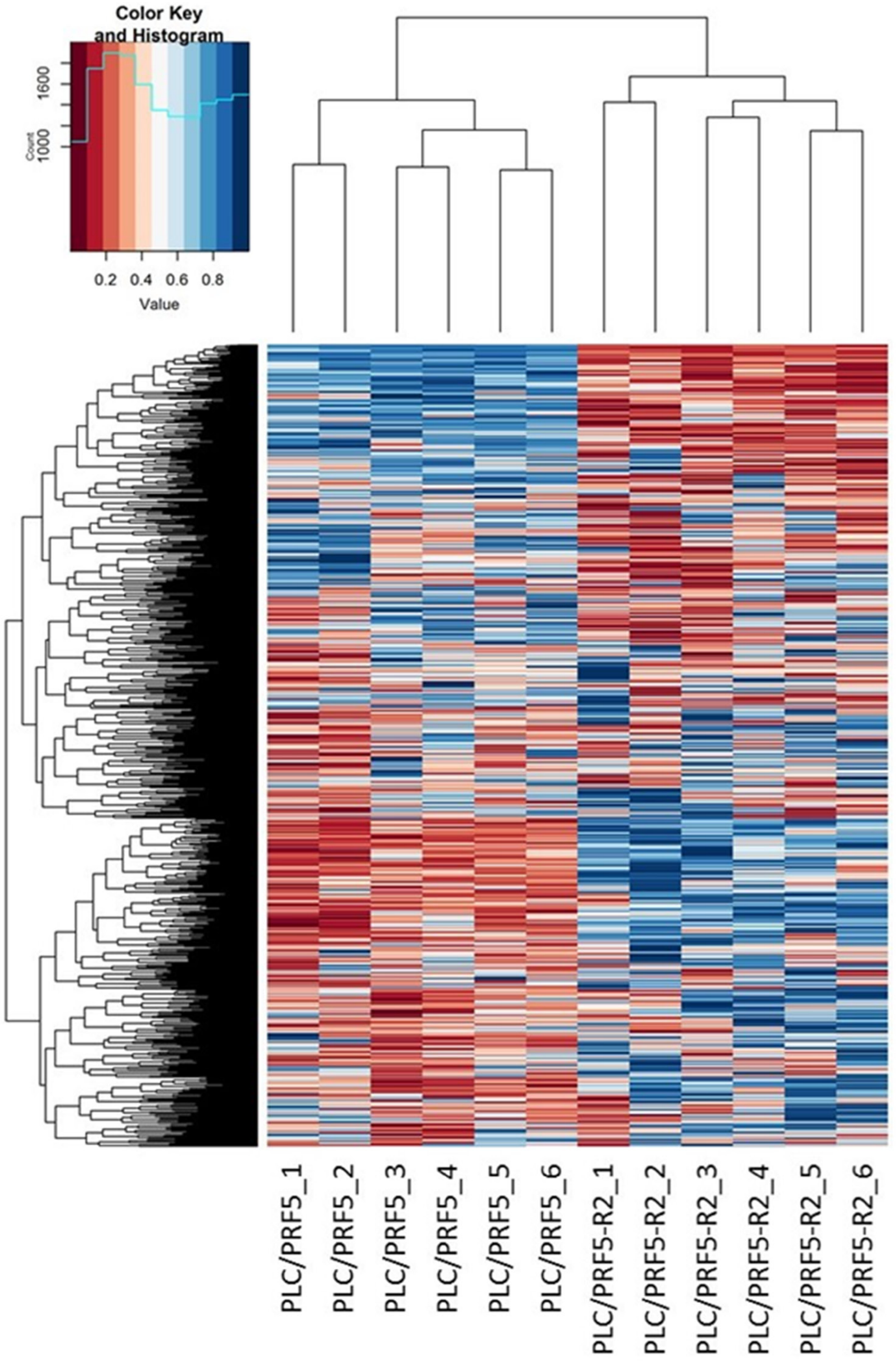

Supplementary Figure 4: Heatmap of array proteins per cluster with significantly altered degree of phosphorylation in PLC/PRF5 cells or PLC/PRF5-R2 cells treated with lenvatinib. A heatmap of array proteins per cluster with significantly altered degree of phosphorylation following incubation with lysates from PLC/PRF5 cells or PLC/PRF5-R2 cells treated with LEN for 48 h was generated from the degree of phosphorylation of 1205 proteins simultaneously measured by the array.

Supplementary Table 1: Clinical response of 53 patients with unresectable hepatocellular carcinoma treated with lenvatinib

**Evaluation (mRECIST)**	***n* (%)<br>(*n* = 53)**
Complete response	2 (3.8)
Partial response	24 (45.3)
Stable disease	25 (47.2)
Progressive disease	2 (3.8)
Objective responce rate (%)	49.1%
Disease control rate (%)	96.2%

mRECIST modified response evaluation criteria in solid tumors.

Supplementary Table 2: List of 63 FRS2-related proteins among all 377 pathways involved in signal transduction. See Supplementary Table 2
